# Supplementary material for: Structure-Based Stabilization of SOSIP Env Enhances Recombinant Ectodomain Durability and Yield
Source: J Virol. 2023 Jan 12;97(1):e01673-22. doi: 10.1128/jvi.01673-22 (PMC9888283; doi:10.1128/jvi.01673-22)
Supplement: Supplemental file 1 — Fig. S1 to S7 and Tables S1 to S3. Download jvi.01673-22-s0001.pdf, PDF file, 8.8 MB [file jvi.01673-22-s0001.pdf]

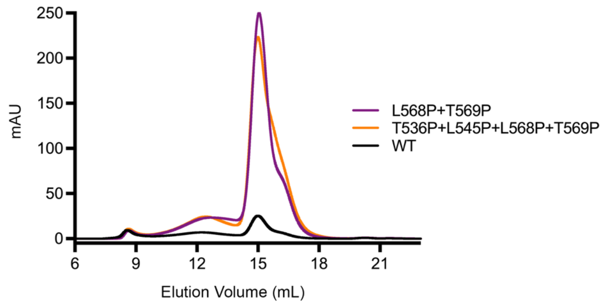

Supplementary Figure 1: **Evaluating effects of quadruple proline-substitution on Env homogeneity and yield**. Size-exclusion chromatograms from a Superose 6 Increase column are shown. CH848 10.17DT DS-SOSIP is colored black (labeled "WT"), CH848 10.17DT DS-SOSIP L568P+T569P is colored purple and CH848 10.17DT DS-SOSIP T536P+L545P+L568P+T569P is colored orange. The curves from CH848 10.17DT DS-SOSIP and CH848 10.17DT DS-SOSIP L568P+T569P are the same as those shown in **Fig. 1C**.

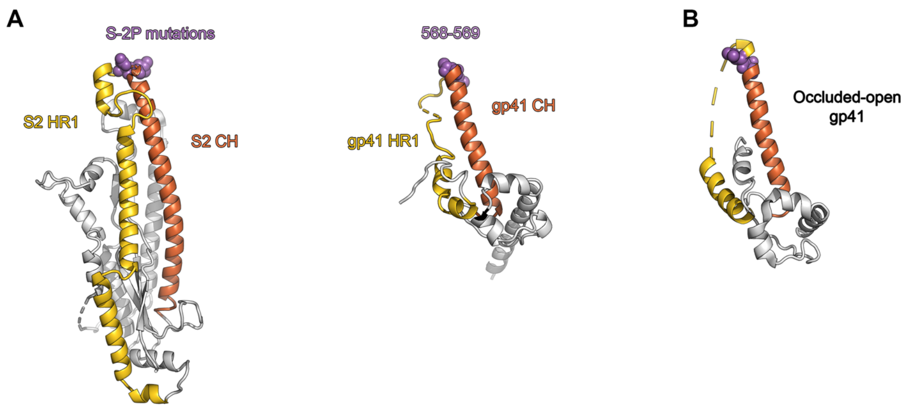

Supplementary Figure 2: Structural comparison of CoV S-2P mutations and Env 2P mutations. (**A**, *left*) A monomer of the S2 subunit of the HCoV-HKU1 Spike in the prefusion conformation (PDB ID: 5I08) is shown as a ribbon diagram, with the heptad repeat 1 (HR1) colored yellow, the central helix (CH) colored orange, and the residues that are altered in the S-2P mutations shown as purple spheres. (**A**, *right*) A monomer of the gp41 subunit in the prefusion conformation (PDB ID: 6VZI) is shown as a ribbon diagram, with the HR1 colored yellow, the CH colored orange, and residues 568-569 shown as purple spheres. (**B**) A monomer of the gp41 subunit in the occluded-open state (PDB ID: 6CM3) is shown as a ribbon diagram, colored according to panel **A**.

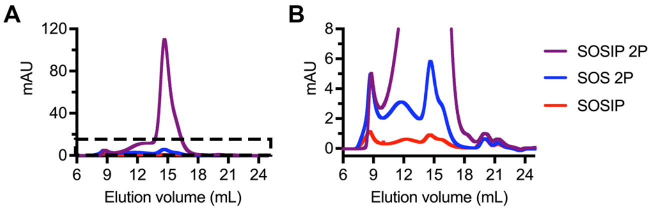

Supplementary Figure 3: The I559P mutation and the 2P mutations act synergistically to boost CH848 10.17DT Env yield. (**A**) Size-exclusion chromatograms from a Superose 6 Increase column are shown. CH848 10.17DT DS-SOSIP is colored red ("SOSIP"), CH848 10.17DT DS A501C+T605C+2P is colored blue ("SOS 2P") and CH848 10.17DT DS-SOSIP-2P is colored purple ("SOSIP 2P"). The dashed box denotes the zoomed-in view that is shown in panel **B**.

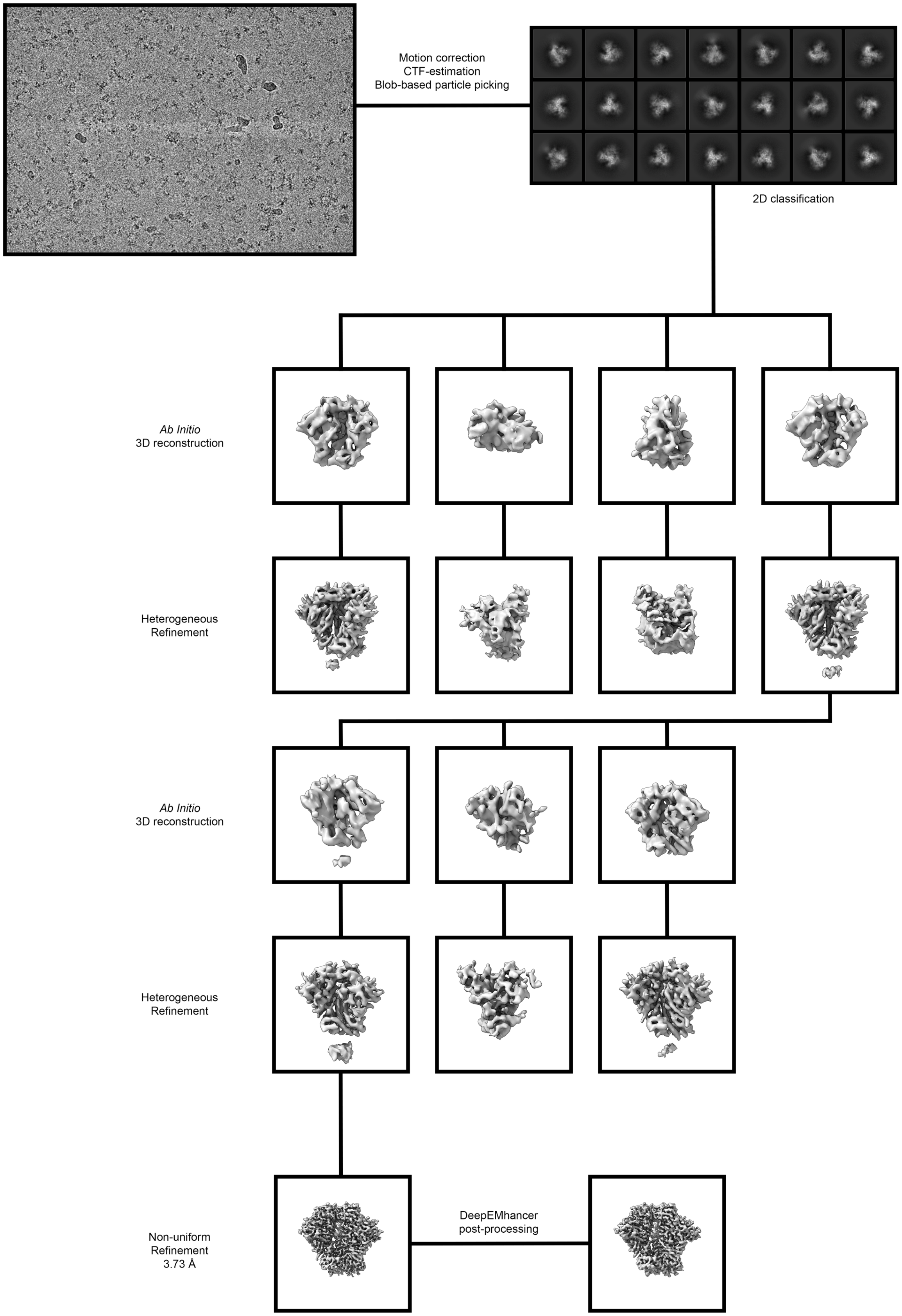

Supplementary Figure 4: Cryo-EM data processing workflow.

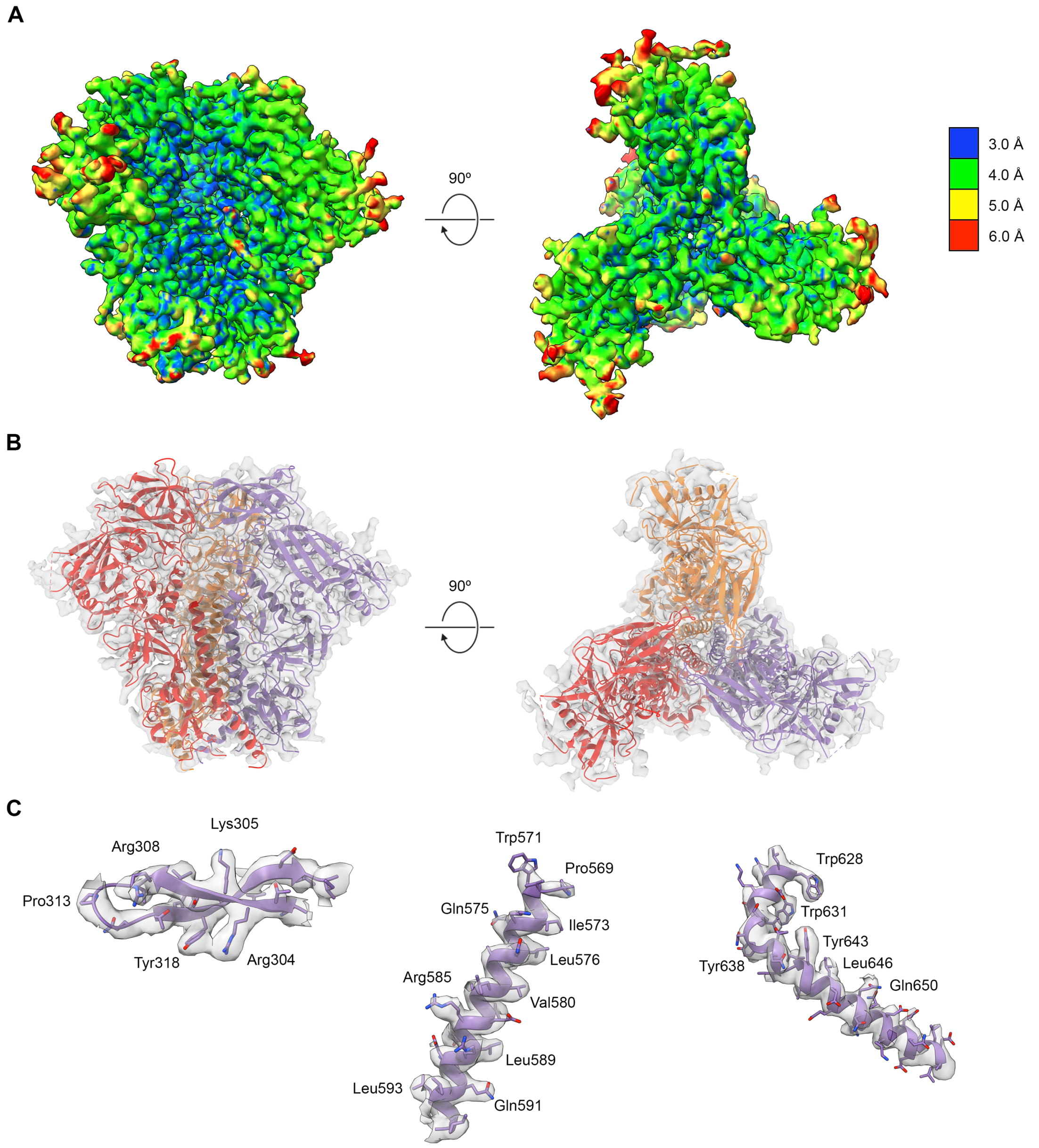

Supplementary Figure 5: Cryo-EM validation. (**A**) Side (*left*) and top (*right*) views of the 3.73 Å CH848 10.17DT DS-SOSIP-2P reconstruction are shown, colored according to local resolution. (**B**) The cryo-EM map is shown as a transparent surface with the corresponding atomic model shown as a ribbon diagram, colored by protomer. (**C**) Portions of the cryo-EM map are shown as a transparent surface with the atomic model shown as a purple ribbon diagram. Side chains are shown as sticks with oxygen atoms colored red and nitrogen atoms colored blue.

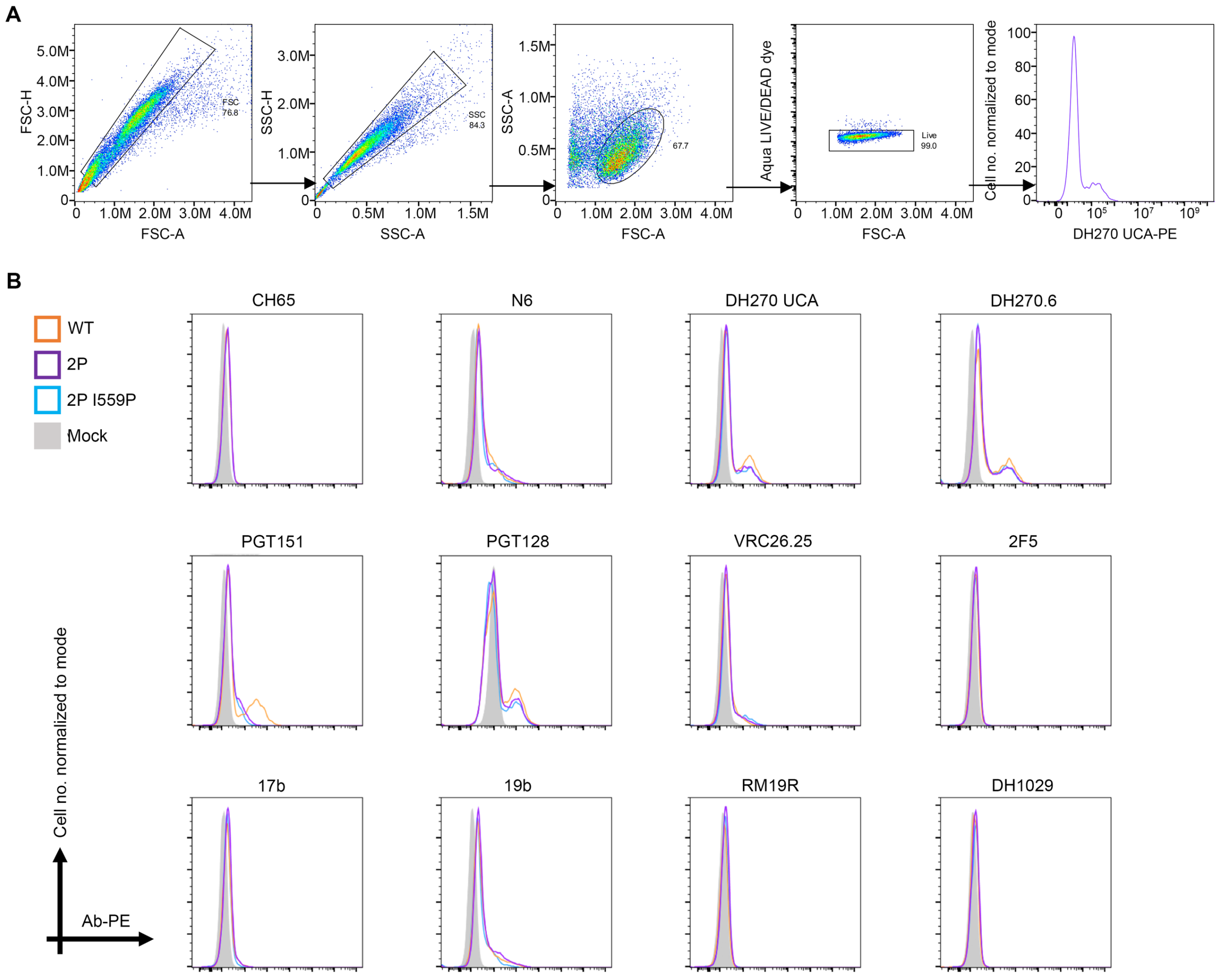

Supplementary Figure 6: Flow cytometric analysis of proline-stabilized CH848 10.17DT gp160s. (**A**) Transiently transfected 293-F cells were gated to exclude doublets, debris and dead cells. mAb binding was detected using a PE-labeled goat anti-human IgG Fc secondary antibody. (**B**) Raw histograms for each mAb that is displayed in **Figure 4A** are shown. CH848 10.17DT gp160 curves are colored orange, CH848 10.17DT 2P gp160 curves are colored purple, CH848 10.17DT 2P I559P gp160 curves are colored blue and untransfected curves are colored gray. The number of cells, normalized to mode, is plotted on the y-axis and PE signal is plotted on the x-axis.

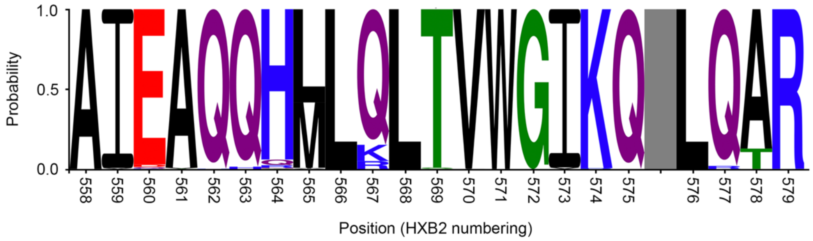

Supplementary Figure 7: Conservation of residues Leu568 and Thr569. A WebLogo plot, calculated using 6,599 Env sequences curated in the LANL HIV database, is shown for positions 558-579. Residues have been colored according to their chemical properties (polar = green, neutral = purple, basic = blue, acidic = red, hydrophobic = black).

**cryo-EM Data Collection**	
Microscope	Titan Krios
Voltage (kV)	300
Detector	Gatan K3
Pixel size (Å/pix)	1.08
Exposure rate (e-/pix/sec)	19.3
Frames per exposure	60
Exposure ($e^-/Å^2$)	61
Defocus range (µm)	-0.8 – -2.4
Micrographs used	5,657
Particles extracted/final	5,780,212/111,026
Symmetry	C3
Resolution (Å) by FSC	
Unmasked 0.5	4.46
Masked 0.5	4.10
Unmasked 0.143	4.10
Masked 0.143	3.73
EMDB ID	28608
**Model Refinement and Validation Statistics**	
Composition	
Amino acids	1692
Ligands (NAG)	63
RMSD Bonds	
Length (Å)	0.012
Angles (°)	1.88
Ramachandran plot	
Outliers (%)	0.0
Allowed (%)	5.4
Favored (%)	94.6
Rotamer outliers (%)	0.47
Cβ outliers (%)	0.0
Clash score	3.29
MolProbity score	1.49
EMRinger score	3.31
PDB ID	8EU8

Supplementary Table 1: Cryo-EM collection and refinement statistics.

Env	Incubation Period (hr)	AUC 0.00-13.80 mL	13.80-16.25 mL	16.25-25.00 mL
CH848 10.17DT DS-SOSIP	0	8.41%	88.31%	3.28%
	48	25.82%	69.63%	4.55%
	96	26.37%	66.69%	6.95%
CH848 10.17DT DS-SOSIP-2P	0	11.45%	85.26%	3.29%
	48	16.07%	80.18%	3.75%
	96	20.10%	75.37%	4.52%

Supplementary Table 2: Area under the curve measurements for SEC forced degradation assay.

Abbreviated Name	Full Name	Clade/Subgroup	GenBank Accession Number	Additional mutations (excluding 2P)
CH848 10.17DT	CH848.3.d0949.10.17	C	KX217749	SOSIP.664*, DS†, DT (N133D+N138T), chimeric BG505 gp41‡
CAM13K	SIVcpzCAM13K	SIVcpz	AY169968	SOSIP.664, DS, Q171K
B41	9032-08.A1.4685	B	EU576114.1	SOSIP.664
JRFL	JRFL	B	U63632.1	SOSIPv6§
T250-4	T250-4	02_AG	MW507842	SOSIP.664, DS
CH505	CH505w24	C	n/a	SOSIP.664, DS, F14#

*SOSIP.664 = A501C+T605C+I559P, R6 optimization of furin cleavage site, truncation after residue 664

†DS = I201C+A433C

‡chimeric BG505 gp41 = Beginning at position 490, residues from BG505 Env were substituted for the native Env sequence

§SOSIPv6 = SOSIP.664 + E64K + A316W + A73C + A561C + E49C + L555C

#F14 = V68I+A204V+V208L+V255L

Supplementary Table 3: Env panel characteristics.
